# Pulmonary artery dissection with patent ductus arteriosus presenting with differential cyanosis: a case report and review of literature

**DOI:** 10.3389/fcvm.2026.1853793

**Published:** 2026-08-14

**Authors:** YeFu Liu, Dou Yuan

**Affiliations:** 1 Department of Cardiology, Chengdu Shang Jin Nan Fu Hospital, West China Hospital of Sichuan University, Chengdu, Sichuan, China; 2 Department of Cardiovascular Surgery, Chengdu Shang Jin Nan Fu Hospital, West China Hospital of Sichuan University, Chengdu, Sichuan, China

**Keywords:** conservative management, Eisenmenger syndrome, patent ductus arteriosus, pulmonary hypertension, spontaneous pulmonary artery dissection

## Abstract

Spontaneous pulmonary artery (PA) dissection is an extremely rare and life-threatening complication of chronic pulmonary hypertension, most commonly associated with congenital heart disease. We report a 54-year-old woman who presented with sharp chest pain, progressive dyspnea, and differential cyanosis between the upper and lower extremities. Laboratory evaluation revealed secondary erythrocytosis and elevated NT-proBNP. Transthoracic echocardiography demonstrated a markedly dilated pulmonary trunk, severe pulmonary hypertension, and a large patent ductus arteriosus with bidirectional shunting. Contrast-enhanced computed tomography confirmed spontaneous PA dissection involving the main pulmonary trunk and both pulmonary arteries. Because of established Eisenmenger physiology, surgical repair was not feasible; the patient declined further intervention and was managed conservatively with diuretics, pulmonary vasodilators, and carefully monitored anticoagulation. She was subsequently discharged against medical advice and lost to follow-up. This case highlights differential cyanosis as a bedside marker of right-to-left ductal shunting, summarizes previously reported cases and their outcomes, and discusses the management challenges of this rare condition.

## 1 Introduction

Spontaneous pulmonary artery dissection (spontaneous PA dissection), that is, dissection arising from intrinsic pulmonary arterial wall disease rather than from catheter-based or other iatrogenic injury, is an extremely rare and often fatal cardiovascular complication, first described as a postmortem finding by Walshe in 1,862 (1). Historically, the condition was diagnosed predominantly at autopsy after sudden death or cardiogenic shock; however, with advances in non-invasive imaging, particularly computed tomography angiography and magnetic resonance imaging, antemortem diagnosis has become increasingly feasible (1, 2).

The condition is intimately linked to chronic pulmonary arterial hypertension, which induces intimal thickening, medial hypertrophy, fragmentation of elastic fibers, atherosclerosis, and cystic medial necrosis, progressively weakening the arterial wall (3, 4). Among the underlying etiologies, uncorrected congenital left-to-right shunts—particularly patent ductus arteriosus (PDA)—are especially important: chronic volume and pressure overload of the pulmonary circulation leads to pulmonary vascular remodeling, shunt reversal, and Eisenmenger syndrome with differential cyanosis (1, 5). Pulmonary artery dissection arising in this setting carries an extremely poor prognosis, with limited therapeutic options and high mortality regardless of treatment approach (6, 7).

We report a rare case of spontaneous PA dissection associated with a large PDA in a middle-aged woman presenting with the distinctive finding of differential cyanosis, and we review previously reported cases to summarize the diagnostic approach, management strategies, and outcomes of this condition.

## 2 Case report

### 2.1 Clinical presentation

A 54-year-old woman presented to the emergency department with sharp, tearing chest pain and progressive dyspnea. The chest pain had commenced one week prior to presentation and had gradually intensified over several days. In the hours preceding admission, she developed acute worsening of dyspnea accompanied by cold sweats and palpitations. There was no history of syncope, hemoptysis, or paroxysmal nocturnal dyspnea, and no prior documented history of congenital heart disease or pulmonary hypertension.

There was no family history of congenital heart disease, sudden cardiac death, or connective tissue disorders. On examination, the patient appeared distressed with tachypnea. Vital signs revealed a heart rate of 106 beats per minute, respiratory rate of 25 breaths per minute, and blood pressure within normal limits. Cardiac auscultation revealed a loud pulmonary component of the second heart sound (P2) without significant murmurs, gallops, or pericardial rub. Pulmonary examination was unremarkable, without crackles or wheezing, and there was no peripheral edema or jugular venous distension. Clubbing of the toes with sparing of the fingers (differential clubbing) was noted, consistent with long-standing differential cyanosis.

The most notable physical finding was differential cyanosis between the upper and lower extremities. Pulse oximetry demonstrated oxygen saturation of 98% in the right upper extremity compared with 85% in the lower extremities. This pattern of differential cyanosis, with lower saturation in the lower body than in the upper body, is pathognomonic for right-to-left shunting through a patent ductus arteriosus in the setting of severe pulmonary hypertension and Eisenmenger physiology (8).

### 2.2 Laboratory investigations

Laboratory evaluation revealed secondary erythrocytosis, with a hemoglobin concentration of 182 g/L and a hematocrit of 0.54, consistent with chronic cyanosis. Arterial blood gas analysis on room air showed pH 7.44, PaO2 56 mmHg, and PaCO2 31 mmHg. NT-proBNP was elevated at 2,360 pg/mL, consistent with right ventricular strain. Cardiac troponin I was within the normal range, and D-dimer was mildly elevated at 0.9 mg/L. Renal and hepatic function, serum electrolytes, and the coagulation profile were otherwise unremarkable. These findings supported chronic hypoxemia with right ventricular strain and helped to exclude acute coronary syndrome as the cause of the chest pain.

### 2.3 Echocardiography

Transthoracic echocardiography demonstrated a markedly dilated main pulmonary artery (approximately 58 mm), right atrial and right ventricular dilatation with right ventricular wall hypertrophy and preserved left ventricular ejection fraction (62%). Moderate tricuspid regurgitation allowed estimation of a right ventricular systolic pressure of approximately 102 mmHg, consistent with severe, near-suprasystemic pulmonary hypertension. Color Doppler imaging demonstrated a large patent ductus arteriosus with bidirectional, predominantly right-to-left shunting. These findings corroborated the invasive hemodynamic measurements described below.

### 2.4 Computed tomography

Emergency contrast-enhanced computed tomography of the chest revealed marked abnormalities of the pulmonary arterial system. The main pulmonary trunk was severely dilated, measuring 57 mm in maximal diameter, consistent with massive pulmonary artery aneurysm formation, and dissection flaps were identified extending into both the right and left pulmonary arteries.

**Figure 1:**
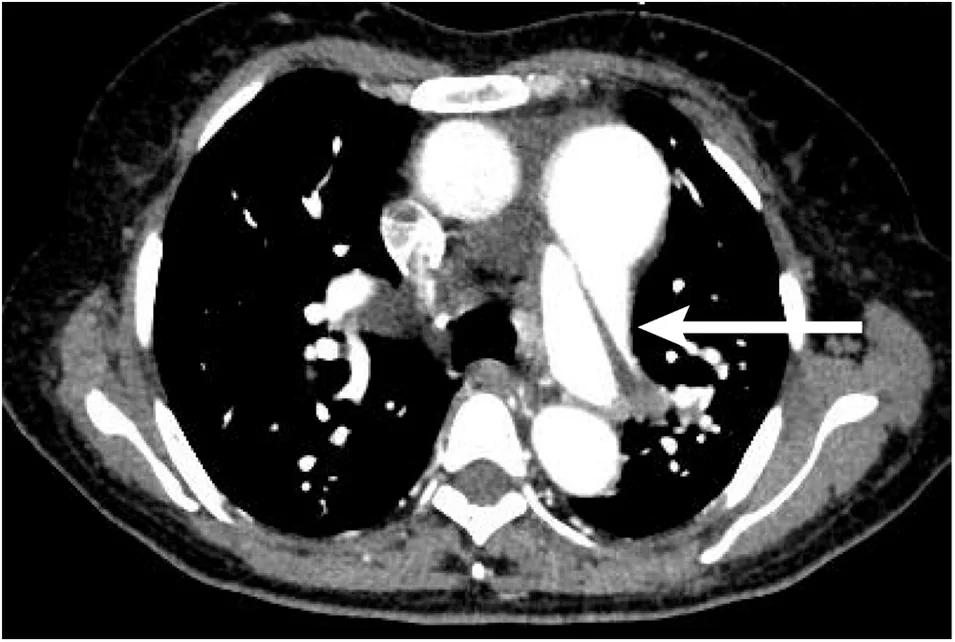
Enhanced CT revealed the left-side pulmonary artery dissection located at the beginning of the left pulmonary artery (arrow).

**Figure 2:**
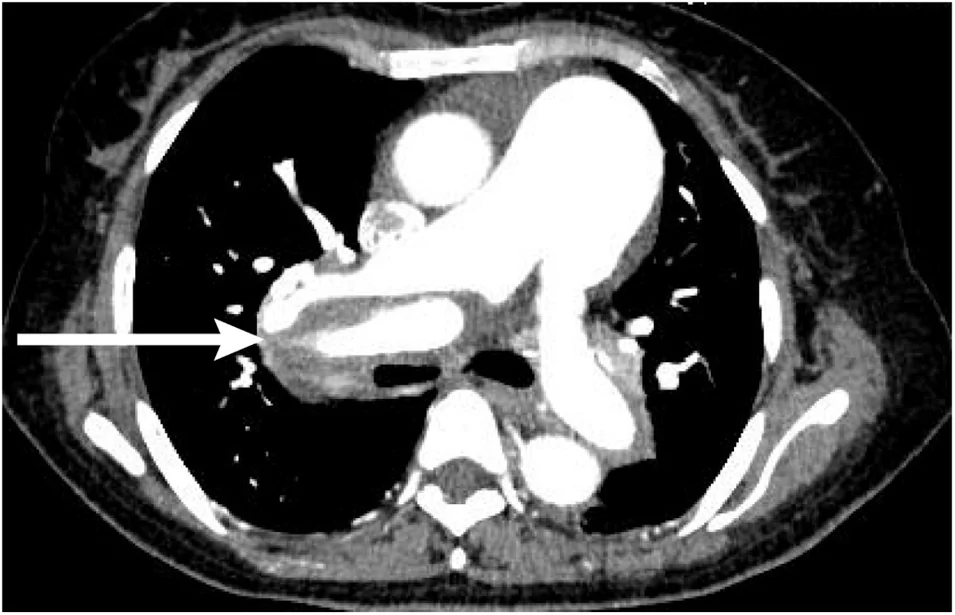
Enhanced CT revealed the right-side pulmonary artery dissection involved the pulmonary trunk and the right pulmonary artery (arrow).

The left-sided dissection originated at the beginning of the left pulmonary artery, with an intimal flap creating true and false lumens (Figure 1). The right-sided dissection was more extensive, involving the pulmonary trunk and extending into the right pulmonary artery (Figure 2). These findings were consistent with bilateral pulmonary artery dissection arising from a markedly dilated, hypertensive pulmonary arterial system.

**Figure 3:**
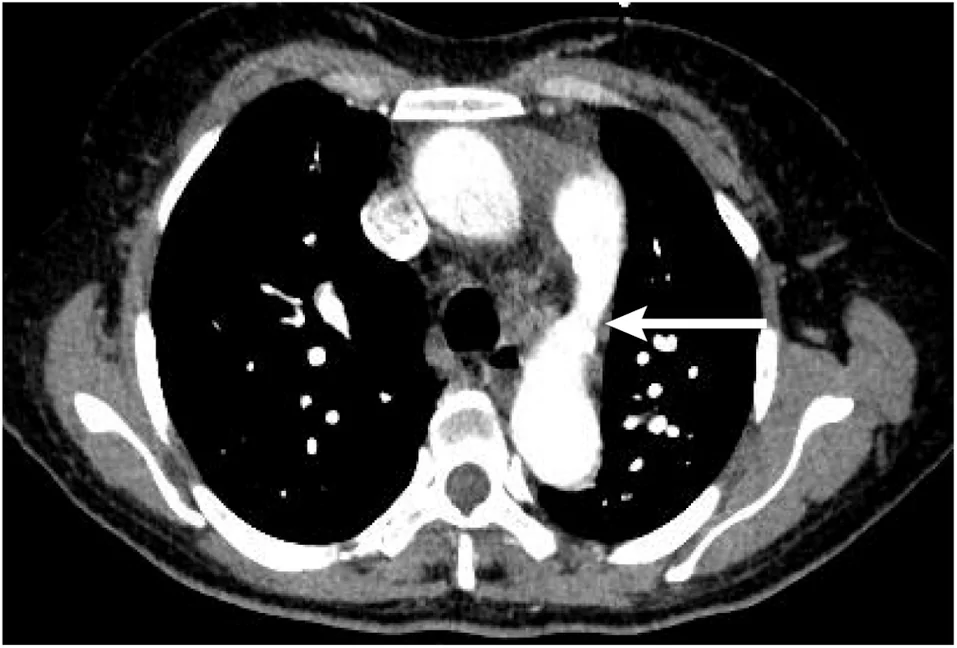
Enhanced CT demonstrated significantly enlarged patent ductus arteriosus connected the left pulmonary artery and the descending aorta (arrow).

Importantly, computed tomography angiography revealed a significantly enlarged patent ductus arteriosus, measuring approximately 14 mm in diameter, connecting the left pulmonary artery to the descending thoracic aorta (Figure 3). The ductus was widely patent, allowing free communication between the aorta and the pulmonary circulation. This anatomical configuration explained the clinical finding of differential cyanosis: deoxygenated blood from the hypertensive pulmonary artery shunted right-to-left through the ductus into the descending aorta, supplying the lower body, while the upper body continued to receive oxygenated blood from the aortic arch.

### 2.5 Hemodynamic assessment

In view of the patient's unstable condition, invasive assessment was deliberately limited to pulmonary artery manometry performed under continuous monitoring in the catheterization laboratory. This confirmed severe pulmonary hypertension with suprasystemic pulmonary artery pressures (approximately 108/52 mmHg, mean 72 mmHg, vs. a systemic arterial pressure of 105/64 mmHg) and demonstrated bidirectional shunting through the patent ductus arteriosus, with predominant right-to-left flow accounting for the observed differential cyanosis. Echocardiographic estimation of right ventricular systolic pressure corroborated these measurements. A complete right heart catheterization with full oximetry runs and vasoreactivity testing was not performed, because catheter manipulation within a dissected, aneurysmal pulmonary trunk in an unstable patient was judged to carry an unacceptable risk of dissection extension or rupture. It should be emphasized that the dissection was documented by computed tomography before any endovascular instrumentation, confirming its spontaneous rather than iatrogenic or catheter-induced origin.

### 2.6 Clinical course and management

The leading differential diagnoses—pulmonary embolism, acute aortic dissection, and acute coronary syndrome—were systematically excluded: computed tomography angiography showed no intraluminal filling defects in the segmental pulmonary arteries and an intact thoracic aorta, while serial cardiac troponin measurements and electrocardiography showed no evidence of myocardial ischemia. The chest pain was therefore attributed to acute expansion of the dissected, aneurysmal pulmonary arterial wall.

The patient was admitted to the intensive care unit for close hemodynamic monitoring and medical optimization. After multidisciplinary discussion, surgical repair was considered technically possible but was deemed to carry prohibitive risk: established Eisenmenger physiology with severe, fixed elevation of pulmonary vascular resistance precluded simple closure of the ductus, and reconstruction of the dissected pulmonary trunk alone would not have corrected the underlying pulmonary vascular disease. Heart–lung transplantation, the only potentially definitive treatment, was discussed with the patient and her family; it was not pursued because of the patient's preferences, her unstable clinical condition, and limited organ availability. She was managed conservatively with oxygen, diuretics, and pulmonary vasodilators.

Anticoagulation with low-molecular-weight heparin was initiated at presentation, when pulmonary embolism remained among the leading differential diagnoses, and was continued at a monitored, weight-adjusted dose after multidisciplinary review. This decision balanced the well-documented risk of *in-situ* thrombosis within massively dilated, slow-flow pulmonary arteries in Eisenmenger syndrome against the potential hazard of bleeding into the false lumen of the dissection; serial imaging during admission showed no extension of the dissection.

After stabilization of her condition, the patient elected to discharge herself from the hospital against medical advice, despite repeated counseling regarding the risks of dissection progression and rupture. Attempts to contact the patient by telephone after discharge were unsuccessful, and she was lost to follow-up; her long-term outcome therefore remains unknown.

## 3 Discussion

### 3.1 Pathophysiology and etiology

Pulmonary artery dissection represents the culmination of chronic hemodynamic stress on the pulmonary arterial wall. According to the law of Laplace, wall stress is directly proportional to intraluminal pressure and vessel radius and inversely proportional to wall thickness; in chronic pulmonary hypertension, elevated pressure combined with progressive arterial dilation creates mechanical stress that exceeds the structural integrity of the remodeled vessel wall, leading to intimal tear and dissection (4). The histopathological substrate includes medial degeneration with loss of elastic fibers, smooth muscle cell apoptosis, and accumulation of mucopolysaccharides—analogous to the cystic medial necrosis seen in aortic dissection—together with atherosclerotic change in long-standing disease (3).

Patent ductus arteriosus is among the most common congenital heart defects (8). When undiagnosed or untreated, the persistent left-to-right shunt leads to progressive pulmonary vascular disease, shunt reversal, and Eisenmenger syndrome, typically by early adulthood (1, 5). The resulting combination of chronic volume overload, pressure overload, and vascular remodeling creates ideal conditions for aneurysm formation, dissection, and rupture (1).

### 3.2 Pulmonary artery aneurysm and predictors of dissection

Pulmonary artery aneurysm is the principal anatomical substrate for spontaneous PA dissection: in the largest available review, encompassing 150 reported cases, dissection occurred almost invariably at the site of a dilated pulmonary artery, and chronic pulmonary hypertension was the dominant underlying condition (9). Aneurysm diameter is therefore the most frequently cited predictor of dissection, but its interpretation requires caution. In our patient, the main pulmonary trunk measured 57 mm on computed tomography; this value was obtained after dissection had occurred, when pressurization of the false lumen and acute expansion of the dissected wall may have enlarged the apparent diameter, so that the pre-dissection diameter was likely smaller and cannot be reconstructed retrospectively. The same caveat applies to the published literature: virtually all reported diameters were measured at or after the time of dissection, and pre-dissection measurements are rarely documented (1, 4, 9). Consequently, no validated diameter threshold predicts dissection, and dissection has been described across a wide range of aneurysm sizes. Beyond absolute diameter, plausible predictors include the severity and chronicity of pulmonary hypertension, the rate of aneurysm expansion on serial imaging, histological fragility of the wall (medial degeneration and loss of elastic fibers), underlying connective tissue disorders, and the presence of an uncorrected congenital shunt (3, 4, 9).

These considerations bear directly on the controversial question of prophylactic surgery for pulmonary artery aneurysm. Kreibich and colleagues have proposed considering surgical repair for aneurysms exceeding approximately 5.5 cm in diameter, for those showing rapid growth, and for aneurysms accompanied by symptoms, intraluminal thrombus, or compression of adjacent structures (4). The evidence base, however, consists of case reports and small series; operative mortality in patients with severe pulmonary hypertension is substantial, and the natural history of unoperated aneurysms is poorly characterized (4, 9). In patients with established Eisenmenger syndrome, prophylactic aneurysm repair without correction of the underlying pulmonary vascular disease offers no proven benefit, and heart–lung transplantation remains the only definitive option (3, 7). Until prospective data become available, we suggest that asymptomatic pulmonary artery aneurysms be monitored with serial cross-sectional imaging to detect rapid expansion, and that prophylactic intervention be reserved for carefully selected patients after multidisciplinary risk assessment. In our patient, who presented only after dissection had occurred in the setting of established Eisenmenger physiology, prophylactic surgery was no longer a meaningful option.

### 3.3 Clinical presentation and diagnosis

The clinical presentation of pulmonary artery dissection is highly variable and non-specific, which historically contributed to the high rate of postmortem diagnosis. Chest pain may be pleuritic, tearing, or pressure-like, mimicking acute coronary syndrome, pulmonary embolism, or aortic dissection (1, 3). In the present case, these alternatives were systematically excluded by computed tomography angiography, serial troponin measurements, and electrocardiography, and the pain was attributed to the dissection itself—an attribution consistent with reports describing tearing chest pain as the dominant presenting symptom (1). In patients with known pulmonary hypertension or congenital heart disease, acute deterioration without a clear cause should raise suspicion for pulmonary artery dissection (4).

The finding of differential cyanosis in the present case was of particular diagnostic value. Because the ductus arteriosus connects the left pulmonary artery to the descending aorta, right-to-left ductal shunting perfuses the lower body with desaturated blood while the upper body receives normally oxygenated blood from the aortic arch—a hallmark of Eisenmenger physiology in patent ductus arteriosus (8). Non-invasive imaging with contrast-enhanced computed tomography or magnetic resonance angiography has substantially improved the antemortem diagnosis of pulmonary artery dissection, allowing antemortem diagnosis and facilitating management decisions (2). Echocardiography provides complementary information on pulmonary artery dimensions, cardiac function, shunt physiology, and estimated pulmonary pressures, and can occasionally visualize the intimal flap itself (1).

### 3.4 Management strategies and outcomes

The optimal management of pulmonary artery dissection remains uncertain owing to the rarity of the condition and the absence of controlled trials; treatment must be individualized according to the underlying etiology, hemodynamic status, anatomical extent of dissection, and patient comorbidities (4). Conservative management may be appropriate for selected patients with stable hemodynamics or contraindications to surgery, and medical therapy includes pulmonary vasodilators (endothelin receptor antagonists, phosphodiesterase type 5 inhibitors, and prostacyclin analogs), diuretics for right heart failure, and oxygen (4, 6).

The role of anticoagulation in pulmonary artery dissection is controversial and deserves specific comment. On one hand, *in-situ* thrombosis is frequent within the aneurysmal, slow-flow pulmonary arteries of patients with Eisenmenger syndrome, and thrombus formation may further compromise the compromised pulmonary circulation; on the other hand, dissection creates a potential risk of hemorrhage into the false lumen. In our patient, anticoagulation was initiated at presentation when pulmonary embolism was still a leading differential diagnosis and was continued cautiously under imaging surveillance after multidisciplinary risk–benefit assessment (4, 6). We emphasize that this decision was individualized and should not be interpreted as a general recommendation; the risks and benefits must be weighed case by case.

Surgical intervention is generally advocated for patients with large aneurysms or hemodynamic compromise but carries substantial risk (4). Options include pulmonary artery reconstruction with graft interposition, aneurysm resection, and closure of associated shunts when feasible (2). Crucially, in established Eisenmenger syndrome with fixed pulmonary vascular disease, closure of the ductus is contraindicated because the shunt acts as a pressure-relief valve; in such patients, heart–lung transplantation remains the only definitive treatment, although it is limited by organ availability and perioperative mortality (3, 7). These considerations directly shaped the management of our patient, in whom neither isolated surgical repair nor ductal closure offered a reasonable prospect of benefit.

### 3.5 Review of previously reported cases

Because pulmonary artery dissection has a poor and variable prognosis, we summarize representative previously reported cases—particularly those associated with patent ductus arteriosus and Eisenmenger syndrome—together with their management and outcomes in Table 1. Reported outcomes range from sudden death within days of presentation despite medical therapy (6, 10) to durable survival after surgical repair in patients without fixed pulmonary vascular disease (2), successful long-term conservative management (6), and prolonged survival after heart–lung transplantation (7). Collectively, these reports suggest that prognosis is determined less by the dissection itself than by the severity and reversibility of the underlying pulmonary vascular disease: patients with pre-Eisenmenger physiology may benefit from surgical repair, whereas those with established Eisenmenger syndrome are generally confined to medical therapy or transplantation.

**Table 1:** Previously reported cases of pulmonary artery dissection associated with chronic pulmonary hypertension, patent ductus arteriosus, and Eisenmenger syndrome.

Study (year)	Age/sex	Underlying condition	Management	Outcome
Khattar et al. (2005) (1)	NR	Chronic pulmonary hypertension (cor pulmonale due to COPD)	Antemortem diagnosis by echocardiography and CT; conservative	Survived at reporting
Ay et al. (2013) (2)	21/F	Pre-Eisenmenger patent ductus arteriosus	Surgical repair: PDA division, aneurysm resection, Dacron graft interposition	Good postoperative outcome
Malm et al. (2015) (5)	NR	Undiagnosed primary pulmonary hypertension	Surgical repair	Survived; reported with literature review
Kim et al. (2016) (6)	43/F	Eisenmenger syndrome with large patent ductus arteriosus	Conservative (oxygen, endothelin receptor antagonist, PDE5 inhibitor)	Stable at 2-year follow-up
Case summarized in (6, 10)	40/M	Eisenmenger syndrome, uncorrected PDA, bilateral dissection	Medical management	Sudden death within 7 days
Tønder et al. (2004) (7)	39/M	Eisenmenger syndrome with pulmonary artery dissection	Heart–lung transplantation	Alive and employed at 6 years
Present case	54/F	Eisenmenger syndrome, large PDA, bilateral dissection	Conservative; surgery and transplantation declined	Discharged against medical advice; lost to follow-up

PAD, pulmonary artery dissection; PDA, patent ductus arteriosus; COPD, chronic obstructive pulmonary disease; PDE5, phosphodiesterase type 5; NR, not reported. In the present case, as in most published reports, the pulmonary artery diameter was measured after dissection had occurred; pre-dissection diameters are rarely documented.

### 3.6 Limitations

This report has several limitations. First and most importantly, the patient discharged herself against medical advice and was lost to follow-up, so the durability of conservative management and her long-term outcome remain unknown; our unsuccessful attempts to contact her after discharge further limited post-discharge data. Second, although there was no clinical evidence of an inherited connective tissue disorder—no suggestive phenotype and no family history—formal evaluation for connective tissue disease and genetic testing (for example, for FBN1, TGFBR1/2, or ACTA2 variants) was not performed, and an underlying arteriopathy cannot be definitively excluded. Third, hemodynamic characterization relied on limited pulmonary artery manometry and echocardiography rather than complete right heart catheterization with oximetry, reflecting the safety constraints imposed by an unstable patient with a dissected pulmonary trunk. Finally, as a single case report, our observations may not be generalizable.

### 3.7 Implications for clinical practice

This case offers several lessons. First, differential cyanosis remains a valuable bedside sign that can rapidly direct diagnostic attention to right-to-left ductal shunting; in an era of increasing reliance on advanced imaging, careful physical examination continues to provide essential diagnostic clues. Second, the combination of pulmonary artery dissection with patent ductus arteriosus and Eisenmenger physiology represents a particularly high-risk constellation with limited therapeutic options; early recognition allows timely discussion of transplantation before irreversible deterioration. Third, patient autonomy plays a central role in management decisions for this condition: given the high-risk nature of any intervention and the uncertain outcomes, informed patient choice between conservative and aggressive strategies must be respected.

The main learning point for clinicians is that unexplained chest pain in a patient with differential cyanosis or known congenital heart disease should prompt active consideration of pulmonary artery dissection. Prompt computed tomography angiography enables antemortem diagnosis, and early multidisciplinary evaluation—integrating echocardiography, laboratory markers of chronic cyanosis and right heart strain, and careful risk stratification—permits informed, patient-centered management decisions.

## 4 Conclusion

Pulmonary artery dissection with patent ductus arteriosus is a rare, life-threatening condition that presents diagnostic and therapeutic challenges. The finding of differential cyanosis provides an important clinical clue to the underlying Eisenmenger physiology with right-to-left shunt (8), and echocardiography together with computed tomography angiography enables rapid antemortem diagnosis (1, 2). While surgical repair may be feasible in selected patients without severe pulmonary vascular disease (2), those with established Eisenmenger syndrome have limited options and a poor prognosis (6, 7, 10). Conservative management with pulmonary vasodilators and supportive care may be appropriate for patients who decline or are not candidates for surgery (4, 6). Increased awareness of this rare complication may improve recognition and allow more informed, patient-centered management decisions.

## Data Availability

The original contributions presented in the study are included in the article/Supplementary Material, further inquiries can be directed to the corresponding author.
